# Paraneoplastischer subakut kutaner Lupus erythematodes

**DOI:** 10.1007/s00393-020-00926-9

**Published:** 2020-11-24

**Authors:** R. Rezazadegan, B. Koushk-Jalali, T. Kuntz, F. Oellig, C. Tigges, A. Kreuter

**Affiliations:** 1 Klinik für Dermatologie, Venerologie und Allergologie, HELIOS St. Elisabeth Klinik Oberhausen, Universität Witten/Herdecke, Josefstr. 3, 46045 Oberhausen, Deutschland; 2 Institut für Pathologie, Mülheim an der Ruhr, Deutschland

**Keywords:** Kutaner Lupus erythematodes, Subakut kutaner Lupus erythematodes, Hydroxychloroquin, Paraneoplastisches Syndrom, Magenkarzinom, Cutaneous lupus erythematosus, Subacute cutaneous lupus erythematosus, Hydroxychloroquine, Paraneoplastic syndrome, Gastric cancer

## Abstract

Der subakut kutane Lupus erythematodes (SCLE) ist ein Subtyp des kutanen Lupus erythematodes, der sich durch hohe Photosensitivität, Auftreten von anulären oder papulosquamösen Hautveränderungen im Bereich der UV-exponierten Körperregionen, serologischen Nachweis von Anti-Ro/SS-A-Antikörpern und milde systemische Beteiligung wie Arthralgien und Myalgien auszeichnet. Wie bei anderen Formen des kutanen Lupus erythematodes kann auch der SCLE durch bestimmte Triggerfaktoren wie UV-Exposition, Zigarettenrauchen oder Medikamente ausgelöst werden. Rheumatische Erkrankungen wie die Dermatomyositis sind seit Langem als paraneoplastische Syndrome bekannt. In den letzten Jahren wird zunehmend über die Assoziation von SCLE und Tumorerkrankungen publiziert. Wir berichten den Fall einer 78-jährigen Patientin, bei der zeitgleich mit der Entwicklung eines SCLE ein Magenkarzinom diagnostiziert wurde. Bei SCLE-Patienten höheren Alters, ausgedehntem Befall außerhalb der UV-exponierten Körperpartien oder neu aufgetretener B‑Symptomatik sollte das Vorliegen eines paraneoplastischen SCLE in Erwägung gezogen und entsprechende diagnostische Schritte zur Abklärung einer Tumorerkrankung sollten eingeleitet werden.

## Abstract (en)

Subacute cutaneous lupus erythematosus (SCLE) is a subtype of cutaneous lupus erythematosus characterized by high photosensitivity, the occurrence of annular or papulosquamous skin lesions located in body regions exposed to UV light, the presence of anti-Ro/SS‑A antibodies, and mild systemic involvement, such as arthralgia and myalgia. Similar to other subtypes of cutaneous lupus erythematosus, certain trigger factors exist for the development of SCLE, such as exposure to UV light, cigarette smoking and drugs. Rheumatic diseases, such as dermatomyositis, have been known as paraneoplastic syndromes for a long time. In recent years, there has been an accumulation of publications on the association of SCLE with malignant diseases. This article reports the case of a 78-year-old female patient who was diagnosed with the concomitant development of SCLE and gastric carcinoma. In all older patients with SCLE, patients with widespread skin affection outside the UV-exposed body areas or patients with B‑symptoms, the presence of a paraneoplastic SCLE should be considered and appropriate diagnostic steps should be initiated to screen for an associated neoplastic disease.

## Falldarstellung

### Anamnese

Eine 78-jährige Patientin wurde zur Abklärung von seit ca. 6 Monaten bestehenden Hautveränderungen stationär in unsere Klinik eingewiesen. Die Patientin gab an, seither unter starkem Juckreiz zu leiden. Zeitgleich zum Auftreten der Hautveränderungen waren der Patientin eine Gewichtsabnahme von 5 kg sowie vermehrtes Sodbrennen aufgefallen. Zudem beklagte sie neben verstärkt aufgetretener Müdigkeit und Abgeschlagenheit auch Arthralgien und Myalgien im Bereich der Arme.

Als weitere Diagnosen bestanden eine medikamentös gut eingestellte chronisch obstruktive Atemwegserkrankung nach langjährigem Nikotinabusus sowie eine Hypothyreose. Lichtempfindlichkeit, Einnahme neuer Medikamente seit Beginn der Hautveränderungen und das Bestehen einer Sicca-Symptomatik wurden von der Patientin verneint.

### Klinischer Befund

**Figure 1:**
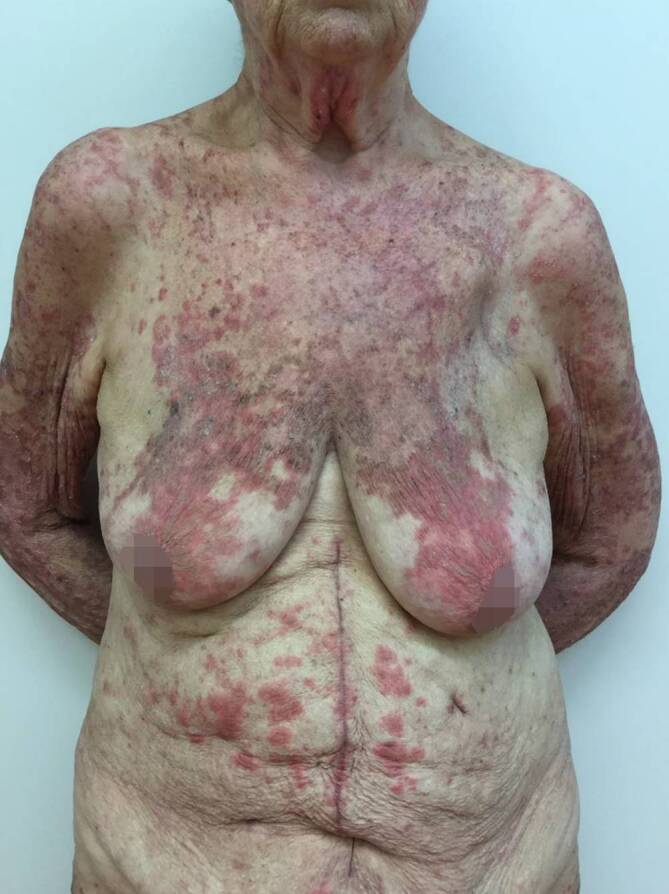

Bei Erstvorstellung in unserer Klinik zeigten sich rötlich-bräunliche, teils papulosquamöse, teils netzförmige, konfluierende Papeln und Plaques, die überwiegend im Bereich des Dekolletés, oberen Rückens, der Brust, des Oberbauches und der Extremitätenstreckseiten lokalisiert waren (Abb. 1 und 2). Das Gesicht sowie die Hände und Füße waren nicht betroffen.

**Figure 2:**
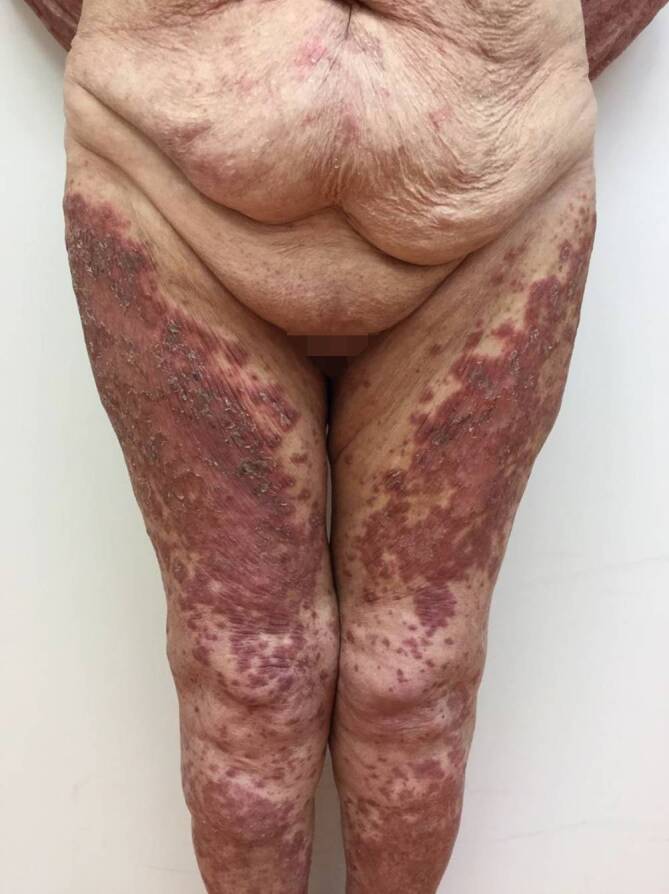

### Histologie

**Figure 3:**
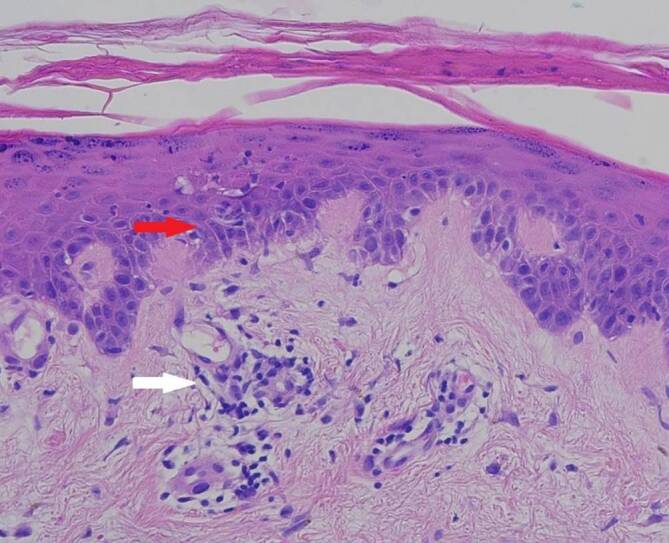

**Figure 4:**
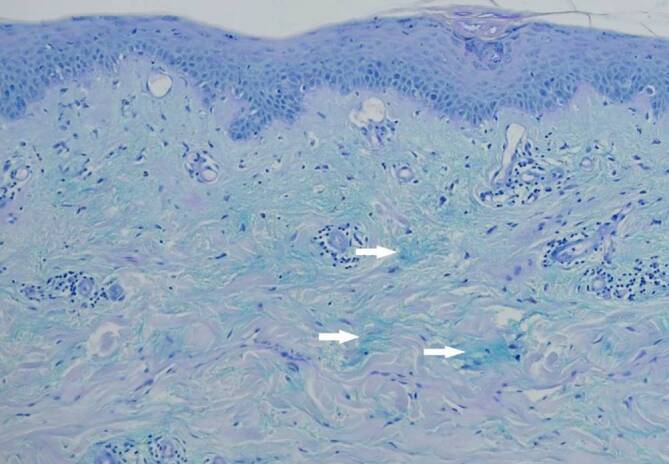

In einer vom Oberbauch entnommenen 4 mm Stanzbiopsie zeigte sich in der Routinefärbung eine manschettenartige, perivaskuläre, überwiegend lymphozytäre Entzündungsreaktion mit fokaler Interfacedermatitis (Abb. 3). In der der PAS-Alcianblau-Färbung fiel in der retikulären Dermis eine vermehrte Muzinablagerung auf (Abb. 4). In der direkten Immunfluoreszenz (Lupusbandtest) zeigte sich eine diskrete granuläre Ablagerung von IgG.

### Weitere Diagnostik

Im Routinelabor zeigten sich abgesehen von einer erhöhten Gamma-Glutamyl-Transferase von 135 U/l (Normbereich: <40 U/l) und einem erniedrigten Hämoglobin von 9,9 g/dl (Normbereich: 12,3–15,3 g/dl) keine abnormen Werte. In der weiterführenden serologischen Autoimmundiagnostik fiel ein leicht erhöhter ANA-Titer von 1:320 (Normbereich: <1:160) auf. Zudem war im Immunoblot auf extrahierbare nukleäre Antikörper der Anti-Ro/SS‑A-Antikörper stark positiv nachweisbar, und es zeigte sich ein erniedrigter Komplementfaktor C3 von 63 mg/dl (Normbereich: 90–170 mg/dl) bei normwertigen C4. Rheumafaktor, Antikörper gegen citrullinierte Proteine und doppelsträngige (ds)DNA sowie Urinstatus waren unauffällig.

In der zur Abklärung einer Photosensitivität bzw. Phototriggerung erfolgten Lichttreppe und Mehrfach-Photoprovokationsdiagnostik mit UV-A- und UV-B-Bestrahlung zeigten sich keine pathologischen Befunde.

In der aufgrund des von der Patientin beschriebenen Sodbrennens durchgeführten Gastroduodenoskopie fiel jedoch im Magen eine suspekte Raumforderung auf, die sich bioptisch als Adenokarzinom des Magens (diffuser Typ nach Laurén) mit endosonographischem Verdacht des Befalls der perigastralen Lymphknoten herausstellte. Die weitere Ausbreitungsdiagnostik (Computertomographie des Thorax und Abdomens sowie Magnetresonanztomographie des Schädels) zeigte keinen Hinweis auf zusätzlich vorhandene Filiae.

### Diagnose

In Zusammenschau von Anamnese, klinischem Bild und Histologie der Hautveränderungen sowie der zusätzlich erhobenen Befunde stellten wir die Diagnose eines subakut kutanen Lupus erythematodes (SCLE), der bei fehlenden „klassischen“ Triggerfaktoren wie Medikamenten und UV-Exposition als paraneoplastische Erkrankung bei Magenkarzinom eingeordnet wurde.

### Therapie und Verlauf

Im Rahmen des stationären Aufenthaltes wurde eine Systemtherapie mit 150 mg Prednisolon intravenös in rasch absteigender Dosierung verabreicht und im weiteren Verlauf oralisiert. Zudem leiteten wir eine systemische Therapie mit 200 mg Hydroxychloroquin 2‑mal täglich ein. Lokaltherapeutisch erfolgte die Behandlung mit Prednicarbat-Creme 1‑mal täglich im Bereich der betroffenen Hautpartien sowie mit pflegenden, harnstoffhaltigen Externa.

Zeitgleich erfolgten eine perioperative Chemotherapie des Magenkarzinoms nach dem FLOT-Schema (bestehend aus den Substanzen 5‑Fluorouracil, Folinsäure, Oxaliplatin und Docetaxel) und eine subtotale Gastrektomie.

**Figure 5:**
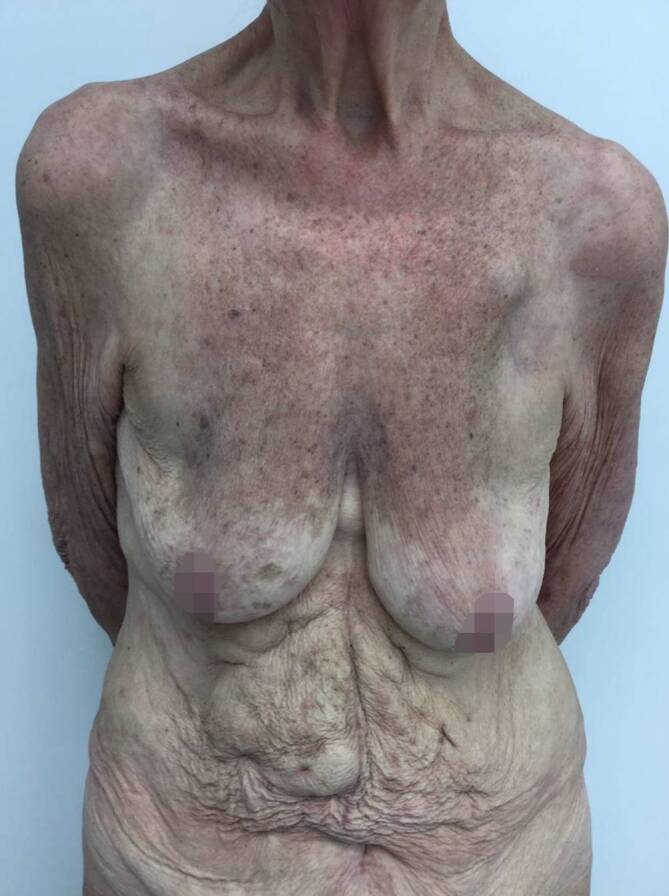

In der ambulanten Verlaufskontrolle zeigte sich bereits nach wenigen Wochen ein deutlich gebesserter Hautbefund, sodass die Therapie mit Hydroxychloroquin 200 mg auf 1‑mal täglich reduziert werden konnte. Vier Monate nach Einleitung der Behandlung mit Hydroxychloroquin waren die Hautveränderungen komplett abgeheilt, es bestand jedoch noch eine residuale postinflammatorische Hyperpigmentierung (Abb. 5 und 6). Die Behandlung mit Hydroxychloroquin wird von der Patientin weiterhin eingenommen, da sich unter der Behandlung auch die Arthralgien und Myalgien deutlich gebessert haben.

**Figure 6:**
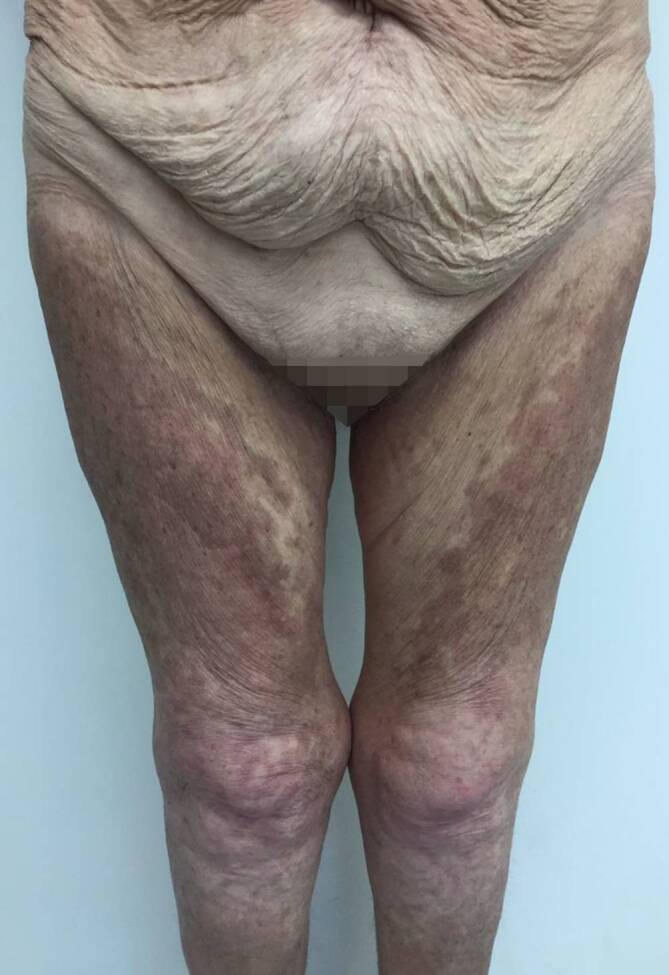

In der onkologischen Nachsorge 12 Monate nach Erstdiagnose ist die Patientin weiterhin tumorfrei.

## Diskussion

Der SCLE wurde erstmalig im Jahr 1979 von Sontheimer als eine Variante des kutanen Lupus erythematodes beschrieben [1]. Es werden 2 klinische Formen des SCLE, ein anulärer und ein papulosquamöser/psoriasiformer Typ, unterschieden. Da sich der SCLE durch eine hohe Photosensitivität auszeichnet, sind die Hautveränderungen meist symmetrisch im Bereich der UV-exponierten Körperpartien lokalisiert. Im Gegensatz zum chronisch diskoiden Lupus erythematodes führt der SCLE nicht zur Vernarbung, kann aber bei Abheilung postinflammatorische Hypo- und Hyperpigmentierungen hinterlassen. Neben den Hautveränderungen können Patienten mit SCLE milde systemische Beschwerden wie Arthralgien und Myalgien haben, eine schwere systemische Organbeteiligung, wie z. B. eine Lupusnephritis, ist jedoch selten [2].

Charakteristisch für den SCLE ist der serologische Nachweis von Anti-Ro/SS-A-Antikörpern (ca. 70–90 %), der stark mit dem Vorhandensein von Photosensitivität korreliert. ANA sind in 60–80 % und Anti-La/SS‑B-Antikörper in 30–50 % der Fälle vorhanden [2]. Histopathologisch zeigen sich beim SCLE eine meist gering ausgeprägte Hyperkeratose neben einem atrophischen Deckepithel, eine vakuolige Degeneration der basalen Epithelzellen (Interfacedermatitis) sowie ein perivaskulär akzentuiertes, lymphozytäres Entzündungsinfiltrat mit dermalen Muzinablagerungen [2].

Wie bei anderen Formen des kutanen Lupus erythematodes existieren auch beim SCLE bestimmte „Triggerfaktoren“ wie ultraviolettes Licht, Zigarettenrauchen und Medikamente. Unter den Subtypen des kutanen Lupus erythematodes wird der SCLE am häufigsten durch Medikamente ausgelöst. Prädisponierende Faktoren für einen medikamenteninduzierten SCLE sind unter anderem ein Sjögren-Syndrom, besondere genetische Konstellationen/Veranlagung (z. B. HLA-B8, -DR3) und/oder Vorhandensein von Anti-Ro/SS‑A-Antikörpern [2]. Beim medikamenteninduzierten SCLE gehen die Hautveränderungen oft über die UV-exponierten Körperregionen hinaus und sind auch an den unteren Extremitäten oder generalisiert vorhanden [3]. Zu den häufigsten Substanzen, die einen SCLE induzieren können, zählen Antimykotika (insbesondere Terbinafin), Diuretika (insbesondere Hydrochlorothiazid) sowie Kalziumkanalblocker. Lokal begrenzte Formen eines SCLE werden primär mit topischen Glukokortikosteroiden oder Calcineurininhibitoren und bei Nichtansprechen mit Hydroxychloroquin behandelt. Bei schwerer und ausgeprägter Hautbeteiligung, wie in unserem Fall vorliegend, sollte zusätzlich zu Hydroxychloroquin der kurzfristige Einsatz systemischer Kortikosteroide in Erwägung gezogen werden. Als Second-line-Therapien können neben Mepacrin (allein oder in Kombination mit Hydroxychloroquin) auch Methotrexat, Retinoide und Dapson eingesetzt werden [2].

Erkrankungen aus dem rheumatischen Formenkreis sind seit Langem als fakultative paraneoplastische Syndrome bekannt [4]. Dies gilt insbesondere für die Dermatomyositis. In den letzten Jahren sind jedoch auch gehäuft Publikationen erschienen, die das Auftreten eines SCLE bei Tumorerkrankungen beschreiben. Die bisher im Zusammenhang mit einem SCLE beschriebenen Tumorentitäten sind überwiegend solide Tumoren wie Mammakarzinom, Kolonkarzinom, Bronchialkarzinom, Ösophaguskarzinom, Uteruskarzinom, Larynxkarzinom oder Leber- und Cholangiokarzinom [5–7]. Dabei kann die jeweilige Tumorerkrankung dem paraneoplastischen SCLE vorausgehen, zeitgleich auftreten oder erst im Laufe der Erkrankung diagnostiziert werden.

Unseres Wissens nach sind bisher 5 Fälle eines SCLE im Rahmen eines Magenkarzinoms beschrieben worden [8–12]. Der genaue pathogenetische Zusammenhang zwischen Tumorerkrankung und paraneoplastischem SCLE ist unklar. Generell werden bei „paraneoplastischen rheumatischen Erkrankungen“ folgende kausale Hypothesen diskutiert [4]:

1. Das Malignom und das paraneoplastische Syndrom haben denselben zugrunde liegenden Auslöser, z. B. eine Virusinfektion.
2. Bestimmte durch den Tumor freigesetzte Mediatoren bzw. inflammatorische Faktoren führen zu einer entzündlichen Reaktion unterschiedlicher Gewebe und Zielstrukturen. Im Fall eines paraneoplastischen SCLE könnte ein dem Anti-Ro/SS‑A-Antikörper ähnliches Tumorantigen als Stimulus für eine photosensitive autoreaktive Entzündungsreaktion fungieren.
3. Die rheumatische Erkrankung ist eine Art von „Hypersensitivitätsreaktion“ auf Proteine oder intrazelluläre Antigene, die vom Malignom produziert werden [4].

Zusammenfassend spricht das zeitgleiche Auftreten von SCLE und Magenkarzinom in dem von uns präsentierten Fall für einen kausalen Zusammenhang, über den in der Literatur bereits wenige Fälle existieren [8–12]. Bei einigen der in der Literatur beschriebenen Fälle eines paraneoplastischen SCLE wurde allein aufgrund der Krankheitsschwere und Ausdehnung der Hautveränderungen das Vorliegen einer Tumorerkrankung in Erwägung gezogen [5, 6]. Dies sollte, wie auch beim medikamenteninduzierten SCLE, insbesondere bei ausgedehntem Hautbefall außerhalb der UV-exponierten Körperpartien erfolgen. Auch bei älteren Patienten mit SCLE ohne „klassische“ auslösende Faktoren wie UV-Exposition, Zigarettenrauchen oder Medikamente und Patienten mit gleichzeitig bestehender B‑Symptomatik sollte an ein zugrunde liegendes Malignom gedacht werden. Aufgrund der zunehmend alternden Bevölkerung könnten Erkrankungen aus dem rheumatischen Formenkreis als fakultative paraneoplastische Syndrome in Zukunft häufiger werden.

## Fazit für die Praxis

- Der SCLE ist ein Subtyp des kutanen Lupus erythematodes, der sich durch hohe Photosensitivität, serologischen Nachweis von Anti-Ro/SS‑A-Antikörpern und milde systemische Beteiligung wie Arthralgien und Myalgien auszeichnet.
- Wie bei anderen Formen des kutanen Lupus erythematodes existieren für den SCLE bestimmte Triggerfaktoren wie UV-Exposition, Zigarettenrauchen und Medikamente.
- Rheumatische Erkrankungen wie die Dermatomyositis sind seit Langem als paraneoplastische Syndrome bekannt.
- In den letzten Jahren wird zunehmend über die Assoziation von SCLE und Tumorerkrankungen berichtet.
- Bei SCLE-Patienten höheren Alters, ausgedehntem Befall außerhalb der UV-exponierten Körperpartien oder neu aufgetretener B‑Symptomatik sollte das Vorliegen eines paraneoplastischen SCLE in Erwägung gezogen werden, und entsprechende diagnostische Schritte zur Abklärung einer Tumorerkrankung sollten eingeleitet werden.
